# Patient-Centered Transition to Clean Intermittent Catheterization After a Sacrectomy for Sacral Chordoma: A Case Report and Narrative Review

**DOI:** 10.7759/cureus.116321

**Published:** 2026-09-15

**Authors:** Irina G Semenenko, Mars Abdaev, Alexander V Bulatov, Yuliy Kubetsky, Dzhamil Rzaev

**Affiliations:** 1 Outpatient Department, Federal Neurosurgical Center, Ministry of Health of the Russian Federation, Novosibirsk, RUS; 2 Spinal Neurosurgery Department, Federal Neurosurgical Center, Ministry of Health of the Russian Federation, Novosibirsk, RUS; 3 Administration and Neurosurgery Department, Federal Neurosurgical Center, Ministry of Health of the Russian Federation, Novosibirsk, RUS

**Keywords:** bladder atony, clean intermittent catheterization, neurogenic bladder, partial sacrectomy, psychological adaptation, sacral chordoma, sacral nerve roots, urinary retention

## Abstract

Sacral chordoma may cause progressive neurological and pelvic organ dysfunction, while definitive surgery can require sacrifice of sacral nerve roots and result in permanent neurogenic bladder. We report a 57-year-old woman with an S2-S4 sacral chordoma presenting with severe lumbosacral pain and voiding dysfunction. Initial imaging demonstrated a 70 × 50 × 42 mm lesion extending into the sacral canal and presacral space. On day three, she underwent a limited decompressive resection of the intraspinal component to relieve root compression and obtain tissue for diagnosis. Histopathology and immunohistochemistry confirmed chordoma. Continued tumor growth was documented, reaching 72 × 51 × 81 mm by week 11. A two-stage procedure was then performed, consisting of endoscopic mobilization of the tumor and pelvic vessels followed by midline S2-S5 sacrectomy. The S1 roots were preserved, whereas the S2-S5 roots were sacrificed. Postoperatively, the patient developed clinically complete bladder atony with painless urinary retention and a bladder volume of 700 mL with 700 mL residual urine. Temporary urethral catheter drainage was used, and an *Escherichia coli* urinary tract infection was successfully treated. The catheter was removed three days before discharge, after which the patient learned and independently performed clean intermittent catheterization. At follow-up, she continued self-catheterization without significant psychological distress. Imaging at month six showed a small stable residual tumor without further growth; proton therapy had not been performed because of a postoperative cerebrospinal fluid collection. This case illustrates that selection of a bladder-emptying strategy after sacral tumor surgery should integrate oncological prognosis, neurological injury, functional status, patient preferences, and structured education. Clean intermittent catheterization can remain feasible even after initial apprehension when training is individualized and supported across inpatient and outpatient care.

## Introduction

Neurogenic lower urinary tract dysfunction (NLUTD) is a clinically significant consequence of lesions involving the spinal cord, conus medullaris, cauda equina, and sacral nerve roots. The principal goals of management are preservation of renal function, maintenance of low-pressure bladder storage, reliable bladder emptying, prevention of urinary tract complications, achievement of continence when feasible, and improvement of quality of life. Current risk-based guidelines emphasize individualized treatment according to the neurological lesion, bladder storage and emptying characteristics, upper urinary tract status, hand function, cognition, caregiver support, and patient preferences [1-3].

Clean intermittent catheterization (CIC) is generally preferred when a patient cannot empty the bladder safely and can perform catheterization independently or with reliable assistance [2-6]. However, even a medically appropriate method may be rejected or used inconsistently when fear, embarrassment, pain, lack of privacy, dependence on others, financial burden, and disruption of work or social activities are not addressed [7-14]. Psychological readiness, structured education, and continued professional support are therefore clinically relevant to treatment adherence and long-term self-management.

Sacral chordoma is a rare, locally aggressive malignant bone tumor arising from notochordal remnants and represents a particularly challenging oncological and functional problem. Because it commonly involves the sacrum and adjacent sacral nerve roots, both the tumor and its surgical treatment can compromise neural pathways responsible for bladder sensation and emptying [15-18]. Adequate tumor removal may require sacrifice of sacral nerve roots involved in bladder sensation, detrusor contraction, sphincter control, bowel function, and sexual function [15-18]. Among the sacral segments, S2-S4 are particularly important for lower urinary tract control. Parasympathetic efferent outflow from S2-S4 travels through the pelvic splanchnic nerves and mediates detrusor contraction, while somatic innervation through the pudendal nerve contributes to voluntary control of the external urethral sphincter. Sacral afferent pathways also convey information related to bladder filling. Consequently, disruption of these neural pathways may impair bladder sensation, detrusor contraction, coordinated emptying, and continence, resulting in neurogenic lower urinary tract dysfunction.

When reliable spontaneous bladder emptying is lost, CIC provides regular bladder drainage while avoiding long-term dependence on an indwelling urethral catheter in patients who are able to perform the technique independently or with assistance [2-6]. Although previous reports have described bladder and bowel outcomes after sacrectomy for sacral tumors [15-18], less attention has been paid to the practical and psychological transition from postoperative catheter dependence to independent CIC. The present case specifically illustrates this transition, including technical training, psychological adaptation, and long-term self-management after sacrifice of the S2-S5 nerve roots. We report the case of a 57-year-old woman with an S2-S4 sacral chordoma who developed clinically complete bladder atony after midline sacrectomy with preservation of the S1 roots and transection of the S2-S5 roots. The patient underwent a staged transition from temporary urethral drainage to independent CIC. This case and the accompanying narrative review illustrate how neurological injury, oncological strategy, physical independence, patient preferences, and psychological adaptation should be integrated when selecting a long-term bladder-emptying method.

## Case presentation

History and initial assessment

A 57-year-old woman had experienced chronic low-back and sacral pain for approximately 20 years. Approximately five months before the index admission, the sacral pain had progressively worsened, and symptoms of voiding dysfunction had developed. Her first hospitalization in the neurosurgical department extended from day zero to day eight.

At admission, she reported lumbar and sacral pain of up to 8/10 on the visual analog scale, pain radiating along the lateral aspect of the right lower extremity of up to 5/10, and subjective leg weakness when walking more than 150 m. Muscle strength was 5/5 in all extremities. Superficial and deep sensation were preserved. Patellar and Achilles reflexes were reduced on the left compared with the right.

Computed tomography (CT) and magnetic resonance imaging (MRI) performed on day zero demonstrated a lytic lesion involving the S2-S4 vertebral bodies, with extension into the sacral ala, sacral canal, and prevertebral space, and destruction of the cortical bone. The lesion measured approximately 70 × 50 × 42 mm. Additional findings included a right median-paramedian L4-L5 disc herniation with grade C spinal canal stenosis, L3-L4 and L5-S1 disc protrusions, and multilevel spondyloarthrosis. Representative preoperative MRI and CT images demonstrating the extent of the sacral tumor are shown in Figure 1.

**Figure 1:**
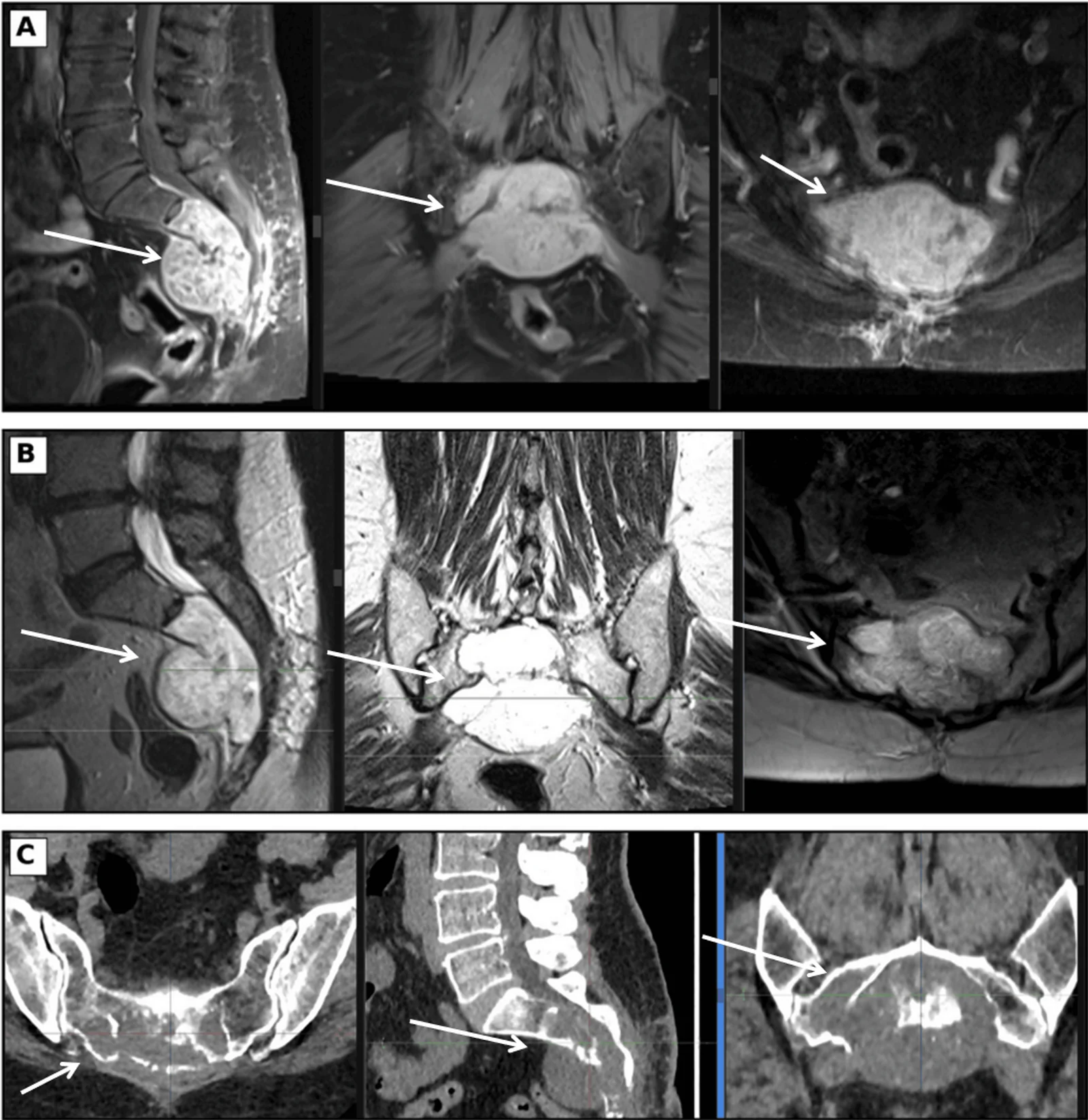
Contrast-enhanced MRI and CT images obtained on day zero, showing a lytic S2-S4 sacral chordoma extending into the sacral canal, sacral ala, and prevertebral space Preoperative imaging during the first hospitalization. (A-B) MRI obtained on day zero demonstrates a lesion involving the S2-S4 vertebral bodies with extension into the sacral ala, sacral canal, and prevertebral space. (C) CT demonstrates a lytic lesion with cortical destruction. The official radiology reports documented dimensions of approximately 70 × 50 × 42 mm. White arrows indicate the sacral tumor and associated sacral involvement.

Before the first operation, bedside bladder scanning demonstrated a bladder volume of 458 mL before voiding and a postvoid residual volume of 62 mL. A strong urge to void was preserved.

First hospitalization and limited decompressive tumor removal

On day three, limited microsurgical partial removal of the intracanal component of the S2-S4 tumor was performed under neurophysiological monitoring. The purpose of the procedure was decompression of the sacral nerve roots and acquisition of tissue for morphologic diagnosis rather than radical tumor removal.

The operation was performed from 14:30 to 16:10, with a duration of one hour 40 minutes and an estimated blood loss of 500 mL. An S2-S4 laminectomy was performed. The tumor occupied the entire sacral canal at this level, was hypervascular, and was densely adherent to the sacral nerve roots. The roots were mobilized and separated from the tumor. The intracanal tumor component was removed piecemeal and to a limited extent within the operative field. A passive drain was placed in the tumor bed.

Contrast-enhanced MRI performed on day four demonstrated persistence of the main tumor mass. At discharge on day eight, no new motor or sensory deficit was present, although voiding dysfunction persisted.

Histopathological and immunohistochemical examinations were performed by an external pathology laboratory. The authors received formal pathology reports, but source microscopic images were not available to the authors for publication. Histologic examination of the specimens obtained during the first operation demonstrated fields and nests of large epithelioid cells with abundant eosinophilic cytoplasm and large oval or elongated centrally located nuclei. Some cells resembled chondrocytes, and occasional atypical mitotic figures were identified. The stroma was sparse and consisted of fibrous septa.

Immunohistochemistry demonstrated positive tumor-cell staining for S-100 and cytokeratin 8, focal positivity for epithelial membrane antigen, and negative staining for cytokeratin 7. The Ki-67 proliferation index reached 10%. The morphologic and immunohistochemical findings were consistent with chordoma, ICD-O 9370/3.

Second hospitalization and continued tumor growth

The patient was readmitted from week 11 to week 15 after the index admission. Pelvic CT performed at week 11 demonstrated a persistent lytic lesion involving the S2-S4 vertebral bodies, measuring approximately 70 × 50 × 42 mm after the limited initial procedure.

Contrast-enhanced MRI performed at week 11 demonstrated continued growth of the residual chordoma to 72 × 51 × 81 mm, with extension into the sacral canal, sacral ala, and prevertebral tissues. The interval radiologic changes after the first procedure and before definitive surgery are shown in Figure 2.

**Figure 2:**
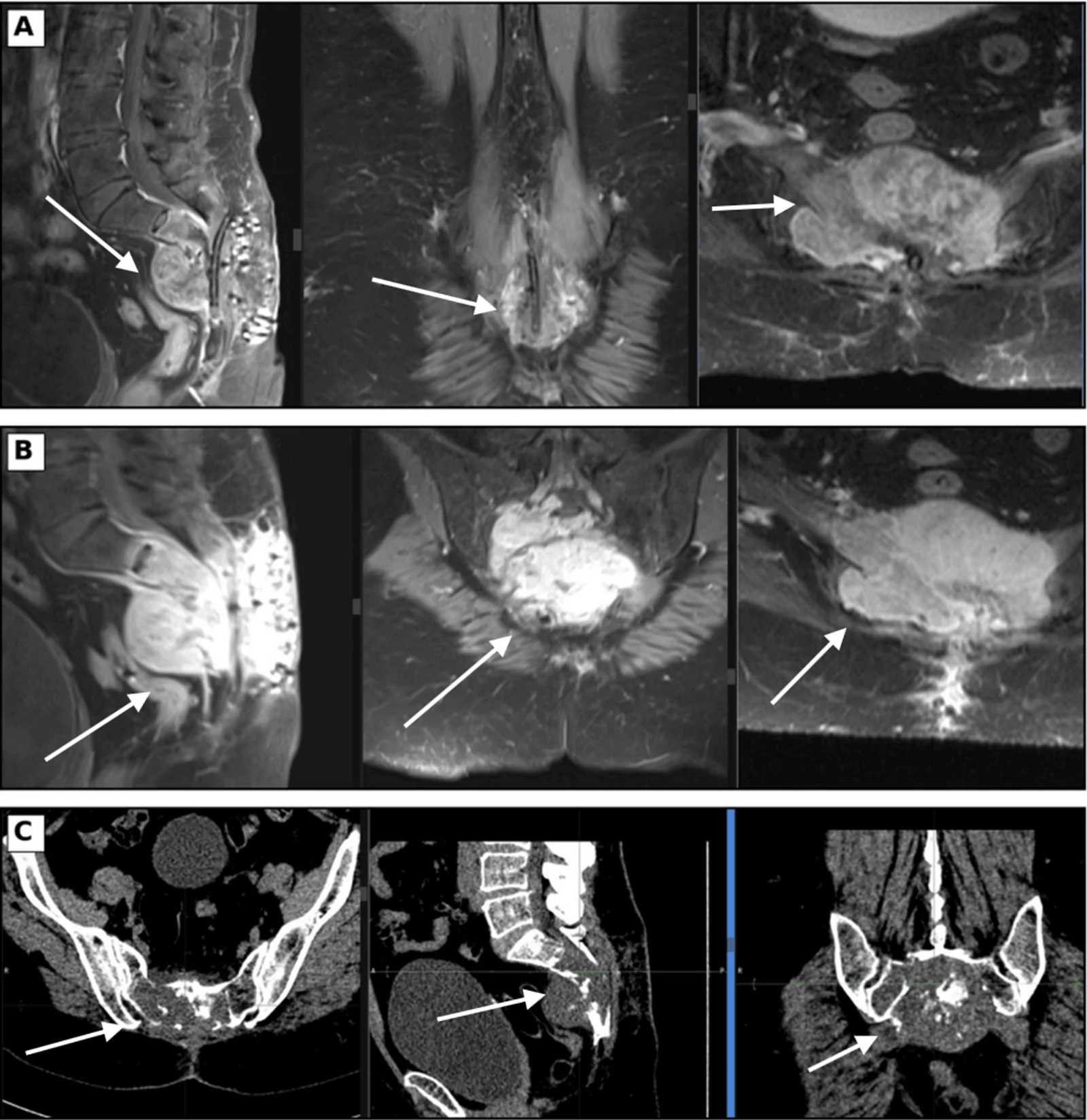
Imaging after the first operation and before the second procedure (A) Contrast-enhanced MRI obtained on day four demonstrates persistence of the main tumor mass after limited decompressive removal. (B) CT obtained at week 11 shows a lytic lesion measuring approximately 70 × 50 × 42 mm. (C) MRI obtained at week 11 documents continued growth to 72 × 51 × 81 mm. White arrows indicate the residual tumor and areas of continued tumor growth.

Two-stage tumor removal and midline sacrectomy

At week 12, a planned two-stage surgical procedure was performed from 10:50 to 18:45. The total operative duration was seven hours 55 minutes, and the estimated blood loss was 1000 mL.

During the first, endoscopic stage, both ureters and the iliac vessels were identified and mobilized. The anterior and lateral surfaces of the tumor were exposed, and the feeding vessels were ligated and divided.

During the second stage, a posterior approach was used. The posterior elements of L4-L5 and the sacrum were exposed, followed by S1 laminectomy and midline S2-S5 sacrectomy. The tumor densely encased the sacral nerve roots and dural sac and occupied the spinal canal at the S3-S5 levels. The dural sac was ligated and transected at the S2 level, and the tumor was removed en bloc.

According to the surgical team, the S1 nerve roots were preserved, whereas the S2, S3, S4, and S5 nerve roots were transected. A near-total resection was achieved, with a small residual tumor focus remaining in the region of a bony ridge, and the patient was referred for consideration of proton therapy.

Postoperative course

The postoperative course included a three-day stay in the intensive care unit. Packed red blood cell transfusions were administered on postoperative days one and five. The patient was mobilized on postoperative day five and initially walked with a frame.

Venous thromboembolism prophylaxis with enoxaparin, analgesic treatment, intravenous fluid therapy, and measures to prevent postoperative ileus were provided. A urinary tract infection caused by *Escherichia coli *was successfully treated with antibiotic therapy.

At discharge, no motor paresis was present. However, anesthesia of the anogenital region and complete urinary retention persisted. Bowel function was preserved. Sexual function was not formally assessed.

Postoperative CT performed on postoperative day one and pelvic MRI performed on postoperative day three demonstrated the sacral resection defect and postoperative changes in the tumor bed. MRI performed on postoperative day 14 showed a fluid-filled postoperative cavity measuring 114 × 109 × 69 mm, subsequently interpreted by the treating team as a postoperative cerebrospinal fluid collection. Representative postoperative CT and MRI findings are shown in Figure 3.

**Figure 3:**
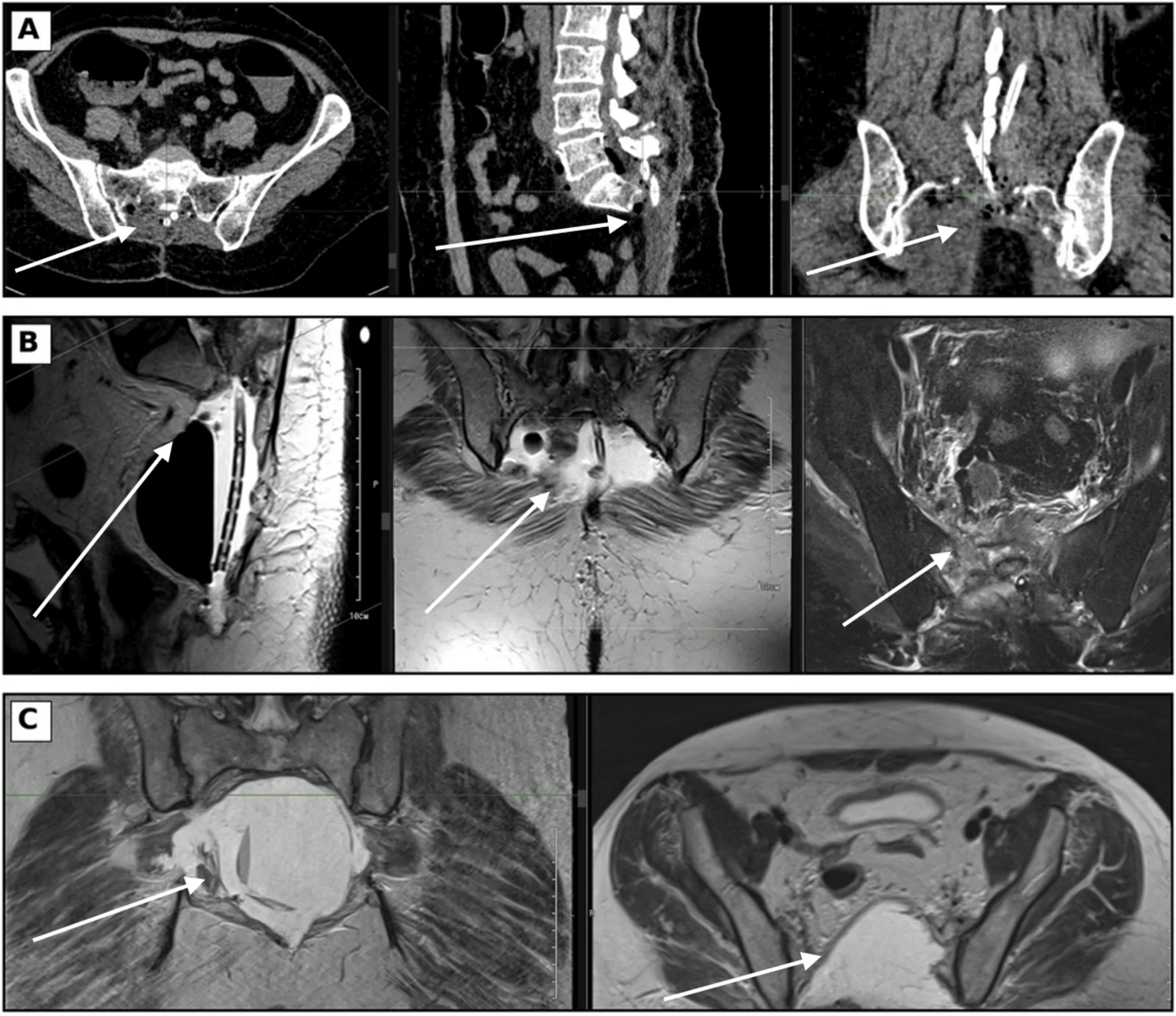
Postoperative imaging after two-stage tumor removal and midline sacrectomy (A) CT obtained on postoperative day one. (B) Pelvic MRI obtained on postoperative day three. (C) Additional postoperative images demonstrate the sacral resection defect and operative bed. MRI obtained on postoperative day 14 showed a fluid-filled postoperative cavity measuring 114 × 109 × 69 mm, subsequently interpreted by the treating team as a postoperative cerebrospinal fluid collection. White arrows indicate the postoperative sacral resection defect and postoperative fluid collection.

Histopathological examination after the second operation

The surgical specimen obtained at week 12 included a 10.0 × 12.0 cm bone fragment containing a gray-white tumor with areas of hemorrhage. This measurement represented the surgical specimen and not the radiologic tumor dimensions.

According to the formal histopathology report, microscopic examination demonstrated fields and nests of large, moderately pleomorphic epithelioid cells with abundant eosinophilic cytoplasm and large oval or elongated centrally located nuclei. Some tumor cells had a chondrocyte-like appearance and were located within a myxoid matrix.

According to the immunohistochemistry report, tumor cells showed positive nuclear staining for brachyury. The Ki-67 proliferation index focally reached 20%. The final diagnosis was chordoma, ICD-O 9370/3.

Postoperative bladder dysfunction

Bladder volume was assessed using a portable BioCon/CUBEScan bladder scanner. Only numerical bladder-volume measurements were recorded in the clinical record; source ultrasound images were not archived. Before the first operation, the prevoid bladder volume was 458 mL, and the postvoid residual volume was 62 mL. Before the second operation, the corresponding measurements were 390 mL and 52 mL. A strong urge to void was preserved during both assessments.

After the second operation, the bladder volume was 700 mL before attempted voiding and remained 700 mL afterward. The patient had no sensation of bladder filling or urge to void. In combination with transection of the S2-S5 nerve roots, these findings were interpreted by the treating urologist as clinically complete bladder atony.

Multichannel urodynamic testing was not performed; therefore, bladder compliance, storage pressure, detrusor activity, and outlet resistance were not formally assessed. Serum creatinine was 73 µmol/L at week 12 and 76 µmol/L at approximately week 14. Dedicated imaging of the upper urinary tract for hydronephrosis was not performed. The overall clinical course and key diagnostic, surgical, and urological events are summarized in Table 1.

**Table 1:** Clinical timeline reconstructed from two discharge summaries, two operative reports, pathology records, radiology reports, and information provided by the treating team

Relative time point	Key clinical event
Approximately month -5	Progressive sacral pain and voiding dysfunction developed.
Day 0	CT and MRI demonstrated an S2-S4 sacral tumor measuring approximately 70 × 50 × 42 mm, extending into the sacral canal, sacral ala, and prevertebral tissues.
Day 3	Limited microsurgical decompression and partial removal of the intracanal tumor component were performed to relieve sacral root compression and obtain tissue for diagnosis.
Week 2	Histopathology and immunohistochemistry confirmed sacral chordoma, ICD-O 9370/3.
Week 11	MRI demonstrated continued growth of the residual tumor to 72 × 51 × 81 mm.
Week 12	Two-stage surgery was performed, including endoscopic tumor and vessel mobilization followed by midline S2-S5 sacrectomy; the S1 roots were preserved and the S2-S5 roots were transected. Postoperatively, clinically complete bladder atony with painless urinary retention developed, and temporary indwelling urethral drainage was initiated.
Week 15	The indwelling catheter was removed three days before discharge. The patient transitioned to CIC six times daily using 14 Fr hydrophilic catheters and achieved independent catheterization by discharge.
Month 6	The small residual tumor remained stable without further growth; proton therapy was not performed because of a postoperative cerebrospinal fluid collection. The patient continued independent clean intermittent catheterization.

Sequential bladder-scan measurements demonstrated preserved bladder sensation and emptying before both operations, followed by complete painless urinary retention after the definitive surgery, as summarized in Table 2.

**Table 2:** Sequential bladder-volume measurements obtained using a portable BioCon bladder scanner The first two assessments demonstrated a preserved urge to void and substantial spontaneous bladder emptying. After the second operation, no urge or spontaneous emptying was present. These measurements were bedside bladder-scan assessments and not formal multichannel urodynamic studies. PVR - postvoid residual

Assessment time point	Bladder volume before voiding (mL)	Bladder volume after attempted voiding / PVR (mL)	Urge to void	Clinical interpretation
Before the first operation	458	62	Strong urge present	Bladder sensation and spontaneous emptying were preserved
Before the second operation	390	52	Strong urge present	Bladder sensation and spontaneous emptying remained preserved
After the second operation	700	700	Absent	Complete painless urinary retention consistent with clinically complete bladder atony

Transition to clean intermittent catheterization

An indwelling urethral catheter was used during the early postoperative period. The catheter was removed three days before discharge, after which the patient transitioned to clean intermittent catheterization (CIC).

The discharge plan recommended CIC six times daily using 14 Ch (14 Fr) hydrophilic catheters, with outpatient follow-up by a urologist. The patient retained normal upper-extremity function, cognition, and general mobility but initially expressed fear of pain, difficulty with positioning, and concern that she would be unable to perform catheterization independently.

Training initially involved assistance from her daughter. Technical explanation and demonstration required approximately five to seven minutes; however, psychological adaptation and acceptance of the method required seven to nine days. These time estimates were based on clinical observation by the treating team rather than formal timing logs or validated psychological questionnaires. By the time of discharge at week 15, the patient was independently performing CIC.

Follow-up

Proton therapy was considered because of the small residual tumor focus but was not performed because of the postoperative cerebrospinal fluid collection. According to follow-up information provided by the treating team, imaging performed at month six demonstrated a stable residual tumor focus without further growth.

At approximately month 12 after the index admission, corresponding to approximately nine months after the definitive operation, the patient continued to perform CIC independently. Reinsertion of an indwelling catheter had not been required. Bowel function remained preserved, while sexual function had not been formally evaluated. The patient reported no symptoms attributable to the residual tumor and no clinically significant psychological distress associated with self-catheterization.

## Discussion

Clinical interpretation and selection of bladder management

Current guidelines for neurogenic lower urinary tract dysfunction emphasize risk stratification and individualized management rather than selection of a bladder-emptying method solely according to the anatomical level of the neurological lesion [1-3]. The same neurological condition may be associated with detrusor overactivity, impaired bladder compliance, detrusor-sphincter dyssynergia, detrusor underactivity, or an acontractile detrusor. Clinical history and postvoid residual volume should therefore be interpreted together with renal function, upper urinary tract imaging, and multichannel urodynamic findings when these results may influence treatment or surveillance.

In the present case, bladder sensation and spontaneous emptying were largely preserved before both operations. The postvoid residual volumes were 62 mL and 52 mL, respectively, and a strong urge to void was present. After midline S2-S5 sacrectomy with preservation of the S1 roots and transection of the S2-S5 roots, the patient had no sensation of bladder filling and was unable to empty a bladder volume of 700 mL. The temporal relationship between surgery and the abrupt loss of sensation and emptying strongly supports a neurogenic cause related to sacral root transection. However, portable bladder scanning measures bladder volume but does not assess detrusor contraction, bladder compliance, storage pressure, or outlet resistance. The term clinically complete bladder atony is therefore appropriate, whereas urodynamically confirmed detrusor acontractility cannot be claimed.

An indwelling urethral catheter was appropriate during the immediate postoperative period because it provided continuous and reliable decompression while the patient was recovering from extensive surgery. For long-term management, clean intermittent catheterization is generally preferred when safe bladder emptying is required, and the patient has adequate cognition, hand function, mobility, anatomical access, or reliable assistance [2-6]. In this patient, preserved upper-extremity function, cognition, and mobility made independent catheterization technically feasible. Removal of the indwelling catheter three days before discharge allowed supervised assessment of her ability to perform the procedure before returning home.

CIC should not, however, be regarded as universally superior for every patient. Its sustainability depends on body positioning, visual and manual ability, privacy, access to suitable catheters, daily schedule, caregiver availability, and the patient's willingness to incorporate the procedure into everyday life. When intermittent catheterization is not feasible, long-term continuous drainage may be required. A suprapubic catheter may be preferable to prolonged urethral catheterization in selected patients, although it remains associated with infection, obstruction, leakage, stone formation, stoma care, and altered body image [2,3,7]. Reflex voiding, the Credé maneuver, and Valsalva voiding should not be used routinely without evidence of urodynamic safety because incomplete emptying or elevated intravesical pressure may place the upper urinary tract at risk [3,6].

Psychological adaptation and self-management

The choice of a bladder-emptying method is not determined by urological safety alone. Qualitative studies involving patients with spinal cord injury have identified anxiety, embarrassment, loss of control, dependence, confidence, independence, and the need for planning as important factors influencing bladder-management decisions [8,9]. Patients may recognize the medical benefits of CIC while simultaneously experiencing fear of pain, concern about urethral injury, shame, reduced privacy, difficulty catheterizing outside the home, or uncertainty about their ability to perform the procedure independently [8-14].

Structured education by trained healthcare professionals can improve confidence, autonomy, and adherence, but a single technical demonstration may be insufficient [10,11,14]. Education should include explanation of the purpose of catheterization, demonstration of the technique, supervised practice, selection of an appropriate catheter, correction of positioning difficulties, discussion of complications, and a clear plan for obtaining supplies and outpatient support. Caregiver assistance may be useful during the initial learning period, but the objective should be to preserve patient autonomy whenever possible.

The course of the present patient illustrates the difference between technical learning and psychological acceptance. The basic explanation and demonstration required only several minutes, whereas normalization of the procedure and confidence in independent catheterization developed over seven to nine days. Initial fear should therefore not automatically be interpreted as refusal or nonadherence. It may represent a predictable stage of adaptation to an intimate and potentially permanent self-care procedure. By discharge, the patient was independently performing CIC, and she continued the method during follow-up without clinically significant psychological distress.

The successful transition was likely supported by several favorable factors: preserved cognition and hand function, the ability to mobilize, involvement of her daughter during the initial training period, repeated inpatient instruction, and the opportunity to practice after removal of the indwelling catheter. These factors should be assessed before concluding that a patient is unable or unwilling to perform CIC.

Sacral chordoma and the functional cost of oncological surgery

Sacral chordomas are slow-growing malignant tumors that may become large before diagnosis and may present with sacral pain, radiculopathy, bowel or bladder dysfunction, sexual dysfunction, or neurological deficits [15]. Surgical treatment requires balancing local tumor control against preservation of sacral nerve roots and pelvic organ function. Sacrectomy is associated with substantial neurological, urological, gastrointestinal, wound-related, and functional morbidity [16-18].

Bladder and bowel outcomes after sacrectomy depend on the level, laterality, and number of preserved sacral roots. Previous studies have shown that preservation of the S3 roots is particularly important for maintaining continence and pelvic organ function [16]. In the present case, the S1 roots were preserved, whereas the S2-S5 roots were transected. This anatomical pattern is consistent with preservation of lower-extremity motor function but profound loss of bladder sensation and detrusor emptying. The postoperative deficit was therefore an anticipated functional consequence of the oncologically necessary operation rather than an isolated catheter-related complication.

Sacral nerve reconstruction has been described in selected patients undergoing resection of sacral chordoma, with reports of recovery of urinary, bowel, and sexual function [17]. Nevertheless, these procedures are technically demanding, are not applicable to every anatomical or oncological situation, and cannot be considered a routine method of preventing postoperative pelvic organ dysfunction. Patients undergoing extensive sacral tumor surgery should therefore receive explicit preoperative counseling regarding the possible need for permanent catheter-based bladder emptying, bowel-management strategies, and changes in sexual function.

Oncological and urological follow-up

A small residual tumor focus remained after the definitive procedure, and proton therapy was considered. Treatment was not performed because of the postoperative cerebrospinal fluid collection. Follow-up imaging at month six demonstrated no further growth of the residual focus, and the patient reported no symptoms attributable to it. Continued oncological surveillance remains necessary because the duration of follow-up is still limited.

Long-term urological surveillance is also required even when CIC is performed successfully. Stable serum creatinine values are reassuring but do not exclude high-pressure storage, impaired bladder compliance, vesicoureteral reflux, or early upper urinary tract dilatation. Periodic assessment should therefore include renal function, bladder-emptying volumes, urinary tract symptoms, and upper urinary tract imaging. Multichannel urodynamic testing should be considered if the results would alter catheterization frequency, pharmacological treatment, surveillance intensity, or decisions regarding additional intervention.

The episode of *Escherichia coli* urinary tract infection was successfully treated during hospitalization. Future bacteriuria should be distinguished from symptomatic infection because catheter users commonly develop bacterial colonization, and unnecessary antibiotic treatment may promote antimicrobial resistance. The patient should also be monitored for urethral trauma, hematuria, increasing difficulty with catheter passage, leakage between catheterizations, and recurrent symptomatic infection.

Practical implications

This case supports several practical principles. First, pelvic organ consequences should be anticipated and discussed before surgical sacrifice of sacral nerve roots. Second, immediate continuous drainage is appropriate when reliable spontaneous emptying cannot be ensured after extensive sacral surgery. Third, the long-term method should be selected according to both urological safety and the patient's functional and psychosocial circumstances. Fourth, initial fear of CIC should prompt repeated education and supervised practice rather than immediate abandonment of the method. Finally, inpatient training should be connected to outpatient urological follow-up because successful catheterization is an ongoing self-management process rather than a single technical lesson.

Limitations

This report has several limitations. It describes a single patient and cannot establish the superiority of CIC over other bladder-management methods. Multichannel urodynamic testing was not performed; therefore, detrusor contractility, bladder compliance, storage pressure, and outlet resistance were not objectively characterized. Dedicated upper urinary tract imaging for hydronephrosis was unavailable, and sexual function was not formally assessed. Psychological adaptation was evaluated clinically rather than with validated questionnaires, and the reported duration of technical and emotional adaptation was based on clinical observation.

The clinical history was reconstructed from two hospitalizations, operative reports, pathology records, radiology reports, and information provided by the treating team. Follow-up remains relatively short, and the status of the residual tumor requires continued imaging surveillance. Despite these limitations, the case provides a detailed temporal association between sacral root transection, loss of bladder sensation and emptying, and successful transition from temporary urethral drainage to independent CIC.

## Conclusions

Sacral chordoma surgery may require sacrifice of the sacral nerve roots and can result in permanent neurogenic lower urinary tract dysfunction. In this patient, preservation of the S1 roots with transection of the S2-S5 roots was followed by loss of bladder sensation and complete painless neurogenic urinary retention. Because multichannel urodynamic testing was not performed, detrusor acontractility or hypotonia could not be formally confirmed. Temporary urethral catheter drainage provided reliable bladder decompression during the early postoperative period, while clean intermittent catheterization became a feasible long-term strategy after structured training and repeated supervised practice.

Selection of a bladder-emptying method should incorporate oncological prognosis, neurological anatomy, renal and urinary tract safety, physical and cognitive function, caregiver availability, patient preferences, and psychological readiness. In this case, preserved cognition, upper-extremity function, mobility, family support, and progressive psychological adaptation facilitated successful transition to independent CIC. Initial fear or difficulty with self-catheterization should not automatically be interpreted as treatment refusal. Individualized education, sufficient time for adaptation, and continued outpatient urological surveillance can support successful long-term management. Long-term surveillance should include assessment of renal function and periodic upper urinary tract imaging, with multichannel urodynamic testing when clinically indicated, to detect potentially silent high-pressure bladder dysfunction or upper urinary tract deterioration.

## References

[1] Ginsberg DA, Boone TB, Cameron AP (2021). The AUA/SUFU guideline on adult neurogenic lower urinary tract dysfunction: diagnosis and evaluation. J Urol.

[2] Ginsberg DA, Boone TB, Cameron AP (2021). The AUA/SUFU guideline on adult neurogenic lower urinary tract dysfunction: treatment and follow-up. J Urol.

[3] European Association of Urology (2026). EAU guidelines on neuro-urology. https://uroweb.org/guidelines/neuro-urology.

[4] Gabbe BJ, Haughton SR, Nunn A (2025). Evaluation of a new model of care for bladder management in a statewide spinal cord service. Spinal Cord.

[5] Gil-Agudo Á, Gutiérrez Henares F, Biscotto S (2025). Monitoring of neurogenic bladder management from hospital admission until discharge in spinal cord injury units: the SIRCA study. Spinal Cord.

[6] Walter J, Wheeler J, Dieter R, Piyevsky B, Khan A (2022). Urological management of the spinal cord-injured patient: suggestions for improving intermittent catheterization and reflex voiding. Uro.

[7] Youssef N, Shepherd A, Best C, Hagen S, Mackay W, Waddell D, El Sebaee H (2023). The quality of life of patients living with a urinary catheter and its associated factors: a cross-sectional study in Egypt. Healthcare (Basel).

[8] Welk B, Myers JB, Kennelly M, McKibbon M, Watson J, Gervais K (2021). A qualitative assessment of psychosocial aspects that play a role in bladder management after spinal cord injury. Spinal Cord.

[9] Welk B, Myers JB, Kennelly M, Elliott CS, McKibbon M, Watson J, Gervais K (2021). Using conjoint analysis to measure the importance of psychosocial traits in the choices of bladder management after spinal cord injury. Neurourol Urodyn.

[10] Blanc BF, Rodríguez-Almagro J, Lorenzo-García C (2021). Quality of life and autonomy in patients with intermittent bladder catheterization trained by specialized nurses. J Clin Med.

[11] Fernandez-Lasquetty Blanc B, Hernández Martínez A, Lorenzo García C (2023). Evolution of quality of life and treatment adherence after one year of intermittent bladder catheterisation in functional urology unit patients. J Clin Med.

[12] Santos-Pérez de la Blanca R, Medina-Polo J, González-Padilla D, Cano-Galán MM, Arrébola-Pajares A, Hernández-Arroyo M, Rodríguez-Antolín A (2023). Evaluation of quality of life and self-reported complications in patients with clean intermittent catheterization: an observational study. J Wound Ostomy Continence Nurs.

[13] Zhang M, Chen Y, Liu J, Luo C, Chen Z, Xu T (2024). Patterns of neurogenic lower urinary tract dysfunction management and associated factors among Chinese community-dwelling individuals with spinal cord injury. Sci Rep.

[14] Zou L, Wu X, Chen S, Feng S, Huang X, Liu W (2026). Self-management challenges and psychosocial support needs in clean intermittent catheterization: a qualitative study of individuals with neurogenic bladder and caregivers. Front Rehabil Sci.

[15] Jelleli N, Mnasser A, Sakly A (2023). Sacral chordoma: an unusual cause of neurogenic bladder - a case report. Ann Med Surg (Lond).

[16] Guo Y, Palmer JL, Shen L (2005). Bowel and bladder continence, wound healing, and functional outcomes in patients who underwent sacrectomy. J Neurosurg Spine.

[17] Berra LV, Armocida D, Palmieri M (2022). Sacral nerves reconstruction after surgical resection of a large sacral chordoma restores the urinary and sexual function and the anal continence. Neurospine.

[18] Branco e Silva M, Martins SCM, Garofo KV, Hanasilo CEH, Etchebehere M (2022). Analysis of morbidity and mortality in patients with primary bone tumors who underwent sacrectomy: a systematic review. J Bone Oncol.

